# Supplementary material for: Mining Salt Tolerance SNP Loci and Prediction of Candidate Genes in the Rice Bud Stage by Genome-Wide Association Analysis
Source: Plants (Basel). 2023 May 30;12(11):2163. doi: 10.3390/plants12112163 (PMC10255615; doi:10.3390/plants12112163)
Supplement: Supplementary file 1 [file plants-12-02163-s001.zip › plants-2403697-supplementary.pdf]

Supplementary Tables

Supplementary Table S1. SNP loci associated with GP, SL and RL in 173 rice accessions in 2020 and 2021.

Trait	QTLs	Chr.	Position	*P* value	R^2 (%)	Year	Cloned genes	Reference
GP	*qGP1-1*	1	23315502	3.62×10^{-4}	7.7	2021		
	qGP1-2	1	38188498	5.28×10^{-5}	12.2	2020		
	qGP2	2	22320650	7.32×10^{-5}	11.8	2020		
	qGP5	5	26737258	2.90×10^{-4}	10.0	2021		
	qGP8	8	19288943	1.28×10^{-4}	9.0	2021		
SL	*qSL2-1*	2	546557	2.50×10^{-4}	8.2	2020	*OsWRKY55* *OsWRKY31*	Xie et al. [56], and Zhang et al. [57]
	qSL2-2	2	796597	2.89×10^{-4}	9.7	2021		
	qSL9-1	9	22686236	1.99×10^{-4}	10.5	2020	*OsISAP1, OsSAP1*	Giri et al. [58]
RL	*qRL1-1*	1	9011153	9.26×10^{-6}	11.9	2021		
	qRL1-2	1	42655740	5.52×10^{-5}	11.8	2021		
	qRL3	3	13222991	5.60×10^{-5}	11.8	2021		
	qRL4-1	4	11933878	2.74×10^{-6}	16.2	2020		
	qRL4-2	4	21289404	3.79×10^{-5}	12.6	2020		
	qRL9-1	9	8687057	4.47×10^{-5}	12.1	2021	*OsMYBc* *OsbZIP71*	Wang et al. [59] Liu et al. [60]

GP: Germination potential; SL: Seedling length; RL: Root length;

56. Xie Z, Zhang ZL, Zou X, Huang J, Ruas P, Thompson D, Shen QJ. Annotations and functional analyses of the rice WRKY gene superfamily reveal positive and negative regulators of abscisic acid signaling in aleurone cells. *Plant Physiol.* **2005**, 137, 176-89; DOI:10.1104/pp.104.054312.
57. Zhang J, Peng Y, Guo Z. Constitutive expression of pathogen-inducible OsWRKY31 enhances disease resistance and affects root growth and auxin response in transgenic rice plants. *Cell Res.* **2008**, 18, 508-21; DOI:10.1038/cr.2007.104.
58. Giri J, Vij S, Dansana PK, Tyagi AK. Rice A20/AN1 zinc-finger containing stress-associated proteins (SAP1/11) and a receptor-like cytoplasmic kinase (OsRLCK253) interact via A20 zinc-finger and confer abiotic stress tolerance in transgenic Arabidopsis plants. *New Phytol.* **2011**, 191, 721-732; DOI: 10.1111/j.1469-8137.2011.03740.x.
59. Wang R, Jing W, Xiao L, Jin Y, Shen L, Zhang W. The Rice High-Affinity Potassium Transporter1;1 Is Involved in Salt Tolerance and Regulated by an MYB-Type Transcription Factor. *Plant Physiol.* **2015**, 168, 1076-90; DOI:10.1104/pp.15.00298.
60. Liu C, Mao B, Ou S, Wang W, Liu L, Wu Y, Chu C, Wang X. OsbZIP71, a bZIP transcription factor, confers salinity and drought tolerance in rice. *Plant Mol Biol.* **2014**, 84, 19-36; DOI: 10.1007/s11103-013-0115-3.

Supplementary Table S2. Gene and functional annotation of SNP loci

QTL	MSU ID	Feature Notes
	LOC_Os02g40590	expressed protein
	LOC_Os02g40600	expressed protein
	LOC_Os02g40610	expressed protein
	LOC_Os02g40620	expressed protein
	LOC_Os02g40630	expressed protein
	LOC_Os02g40640	expressed protein
	LOC_Os02g40650	expressed protein
	LOC_Os02g40664	zinc finger family protein
	LOC_Os02g40680	mis12 protein, expressed
	LOC_Os02g40690	expressed protein
	LOC_Os02g40700	enzyme of the cupin superfamily protein
qGPR2-1	*LOC_Os02g40710*	ammonium transporter protein
	LOC_Os02g40730	ammonium transporter protein
	LOC_Os02g40750	pentatricopeptide domain containing protein
	LOC_Os02g40760	expressed protein
	LOC_Os02g40770	SET domain containing protein
	LOC_Os02g40784	WAX2
	LOC_Os02g40800	expressed protein
	LOC_Os02g40810	Zinc finger, ZZ type domain containing protein
	LOC_Os02g40820	expressed protein
	LOC_Os02g40830	succinyl-CoA ligase beta-chain, mitochondrial precursor
	LOC_Os02g40840	alcohol oxidase

	LOC_Os02g40850	retrotransposon protein
	LOC_Os02g40860	CK1_CaseinKinase_1.5-CK1 includes the casein kinase 1 kinases
	LOC_Os02g40870	phosphatidylinositol N-acetylglucosaminyltransferase subunit C
	LOC_Os02g40880	ribosomal protein L14
	LOC_Os02g40890	GLTP domain containing protein
	LOC_Os02g40900	RNA recognition motif containing protein
	LOC_Os02g40910	hypothetical protein
	LOC_Os02g40920	transposon protein, CACTA, En/Spm sub-class
	LOC_Os09g28240	expressed protein
	LOC_Os09g28250	retrotransposon protein
	LOC_Os09g28260	retrotransposon protein, Ty3-gypsy subclass
	LOC_Os09g28270	retrotransposon protein
	LOC_Os09g28280	gibberellin receptor GID1L2
	LOC_Os09g28290	hypothetical protein
	LOC_Os09g28300	remorin C-terminal domain containing protein
qSLR9	*LOC_Os09g28310*	bZIP transcription factor
	LOC_Os09g28320	expressed protein
	LOC_Os09g28330	transposon protein
	LOC_Os09g28340	expressed protein
	LOC_Os09g28354	CPuORF39 - conserved peptide uORF-containing transcript
	LOC_Os09g28370	retrotransposon protein
	LOC_Os09g28380	hypothetical protein

LOC_Os09g28390	cytochrome P450
LOC_Os09g28400	alpha-amylase precursor
LOC_Os09g28410	expressed protein
LOC_Os09g28420	alpha-amylase precursor
LOC_Os09g28440	AP2 domain containing protein
LOC_Os09g28450	Paramyosin
LOC_Os09g28470	leucine-rich repeat family protein
LOC_Os09g28460	xyloglucan fucosyltransferase
LOC_Os09g28480	expressed protein

Supplementary Table S3. Names and origins of 173 rice accessions used for association mapping.

Code	Germplasm name	Origin	Latitude	Longitude	Germplasm IDa
1	Liuyezhan	Hubei, China	30	114	17-00524
2	Shufeng 101	Sichuan, China	30.39	104.05	ZD-00760
3	Chengnongshuijing	Sichuan, China	30.39	104.05	ZD-03386
4	Guichao 2hao	Guangdong, China	23.08	113.15	H1645
5	Zaoxian 14	Anhui, China	37.51	117.18	11-00670
6	Chenwan 3hao	Hunan, China	28.11	113	ZD-00358
7	Xiangaizao 10hao	Hunan, China	28.11	113	ZD-01402
8	Digludao	Sihong, Jiangsu, China	33.46	118.23	H1312
9	Sujing 353	Suzhou, Jiangsu, China	31.32	120.62	C1511
10	Huaidao 8hao	Huaian, Jiangsu, China	33.5	119.15	SS200410
11	Sihao 4385	Sihong, Jiangsu, China	33.46	118.23	H1315
12	Dongzheng 1640	Hongze, Jiangsu, China	33.28	118.85	C1515
13	Huaidao 9hao	Huaian, Jiangsu, China	33.5	119.15	SS200607
14	Huifeng 2	Yancheng, Jiangsu, China	33.38	120.13	C1509
15	Yandao 9hao	Yancheng, Jiangsu, China	33.38	120.13	SS200506
16	Lianjing 4hao	Lianyungang, Jiangsu, China	34.59	119.16	SS200704
17	Yandao 8hao	Yancheng, Jiangsu, China	33.38	120.13	SS200307
18	Huifeng 1	Yancheng, Jiangsu, China	33.38	120.13	C1508
19	Huaidao 11hao	Huaian, Jiangsu, China	33.5	119.15	SS200805
20	Sihao 4141	Sihong, Jiangsu, China	33.46	118.23	H1332
21	Sihao 4029	Sihong, Jiangsu, China	33.46	118.23	H1333
22	Sihao 4259	Sihong, Jiangsu, China	33.46	118.23	H1334
23	Sihao 4081	Sihong, Jiangsu, China	33.46	118.23	H1337
24	Sihao 4041	Sihong, Jiangsu, China	33.46	118.23	H1338
25	Yandao 6hao	Yancheng, Jiangsu, China	33.38	120.13	SS200205
26	Sihao 4031	Sihong, Jiangsu, China	33.46	118.23	H1340
27	Zhenghan 2hao	Zhengzhou, Henan, China	34.76	113.65	GS2003031
28	Yujing 6hao	Zhengzhou, Henan, China	34.76	113.65	GS980002
29	Cbao	Hefei, Anhui, China	31.86	117.27	H1661
30	Jingnuo 330	Hefei, Anhui, China	31.86	117.27	H1346
31	Wandao 68	Hefei, Anhui, China	31.86	117.27	WPS03010384
32	Wanjingnuo	Hefei, Anhui, China	31.86	117.27	H1653
33	Zhongjing 212	Nanjing, Jiangsu, China	32.04	118.78	WS891061
34	Xiangjing 9407	Nanjing, Jiangsu, China	32.04	118.78	LS891061
35	Dongzhengwuyunjing 21	Hongze, Jiangsu, China	33.28	118.85	SS200705-1
36	Malaihong	Nanjing, Jiangsu, China	32.04	118.78	T050
37	Zhengdao 10hao	Zhenjiang, Jiangsu, China	32.2	119.44	SS200710
38	Huajing 6hao	Huaian, Jiangsu, China	33.5	119.15	SS200706
39	Huajing 5hao	Huaian, Jiangsu, China	33.5	119.15	SS200505
40	Zhongjing 9677	Nanjing, Jiangsu, China	32.04	118.78	C1512
41	Suwujing	Wujin, Jiangsu, China	31.78	119.95	SS201009
42	Wuyunjing 8hao	Wujin, Jiangsu, China	31.78	119.95	SZS313
43	Nannongjing 004	Nanjing, Jiangsu, China	32.04	118.78	T248
44	Ningjinghui 260	Nanjing, Jiangsu, China	32.04	118.78	H1371
45	Nannongjing 1R	Nanjing, Jiangsu, China	32.04	118.78	H1372

46	Yuedao 12	Vietnam	10.22	106.01	Y1A02375
47	Ningjinghui 237	Nanjing, Jiangsu, China	32.04	118.78	H1374
48	Hongmangshajing	Kunshan, Jiangsu, China	31.39	120.95	T630
49	Wumangyedao	Jinshan, Shanghai, China	30.75	121.33	T335
50	Xiaobaidao	Wuxian, Jiangsu, China	31.32	120.62	T208
51	Xiepihuang	Taicang, Jiangsu, China	31.45	121.1	T203
52	Baoxintaihuqing	Wujiang, Jiangsu, China	31.16	120.63	T834
53	Baikenuo	Wujiang, Jiangsu, China	31.16	120.63	T354
54	Shengtangqing	Changshu, Jiangsu, China	31.64	120.74	T759
55	Hongjiaozhan	Wuxian, Jiangsu, China	31.32	120.62	T888
56	Yuedao24(LCV18)	Vietnam	10.22	106.01	
57	Wanhuangdao	Wuxian,Jiangsu,China	31.32	120.62	T815
58	Zaoshirihuangdao	Wuxian, Jiangsu, China	31.32	120.62	T728
59	24248	Nanjing, Jiangsu, China	32.04	118.78	
60	Kangbingyueguang	Japan	35.68	139.69	H1524
61	Youzhiyueguang	Japan	35.68	139.69	Y1A01876
62	Haobuqia	Wuxian, Jiangsu, China	31.32	120.62	21-00357
63	Xu91075	Xuzhou, Jiangsu, China	34.26	117.2	H1418
64	Xudao 25-7	Xuzhou, Jiangsu, China	34.26	117.2	H1419
65	863B	Nanjing, Jiangsu, China	32.04	118.78	H1425
66	9522B	Changzhou, Jiangsu, China	31.79	119.95	GS20000008
67	Yuedao 68	Vietnam	10.22	106.01	Y1A02418
68	Biaojiyongzijing	Nanjing, Jiangsu, China	32.04	118.78	H1427
69	Zigu	Nanjing, Jiangsu, China	32.04	118.78	H1437
70	SH189	Sihong, Jiangsu, China	33.46	118.23	Y1A01866
71	SHN1	Sihong, Jiangsu, China	33.46	118.23	Y1A01867
72	Jiangpuchangliheimidao	Nanjing, Jiangsu, China	32.04	118.78	H1492
73	Chuan 6xian	Chengdu, Sichuan, China	30.67	104.06	H1506
74	Chuan 5xian	Chengdu, Sichuan, China	30.67	104.06	H1507
75	Ludao	Nanjing, Jiangsu, China	32.04	118.78	H1508
76	A7444	Nanjing, Jiangsu, China	32.04	118.78	H1476
77	Qing 7	Yancheng, Jiangsu, China	33.38	120.13	H1471
78	Yuedao 3	Vietnam	10.22	106.01	Y1A02370
79	Longnuo 3hao	Haerbin, Heilongjiang, China	44.04	125.42	HS2009015
80	Longjing 28	Haerbin, Heilongjiang, China	44.04	125.42	HS2009011
81	Longjing 27	Haerbin, Heilongjiang, China	44.04	125.42	HS2009010
82	Longjing 20	Haerbin, Heilongjiang, China	44.04	125.42	HS2007004
83	Mudanjiang 28	Mudanjiang, Heilongjiang, China	44.58	129.6	HS2006006
84	Longjing 22	Haerbin, Heilongjiang, China	44.04	125.42	HS2008010
85	Yuedao 32	Vietnam	10.22	106.01	Y1A02326
86	Longdao 8hao	Haerbin, Heilongjiang, China	44.04	125.42	HS2008019
87	Longdao 6hao	Haerbin, Heilongjiang,	44.04	125.42	HS2006004

		China			
88	Jianongnuo 2hao	Haerbin, Heilongjiang, China	44.04	125.42	H1600
89	Wunuoyihao	Haerbin, Heilongjiang, China	44.04	125.42	H1611
90	Zhongzuo 93	Mudanjiang, Heilongjiang, China	44.6	129.58	JS1995001
91	Xudao 4hao	Xuzhou, Jiangsu, China	34.15	117.11	CNA20040007.X
92	Xudao 5hao	Xuzhou, Jiangsu, China	34.15	117.11	GS2006059
93	Yuedao 37	Vietnam	10.22	106.01	Y1A02397
94	Xudao2hao	Xuzhou, Jiangsu, China	34.15	117.11	
95	Shengdao808	Haerbin, Helongjiang, China	44.04	125.42	
96	Shengdao 14	Jinan, Shandong, China	36.4	117	H1701
97	Yangguang 200	Xuzhou, Jiangsu, China	34.15	117.11	GS2008043
98	Yanjing 8hao	Yancheng, Jiangsu, China	33.38	120.13	ZD-05649
99	Zaijinjing	Songhuajiang, Heilongjiang, China	41.42	119.52	H1614
100	Sihao 4280	Sihong, Jiangsu, China	33.46	118.23	H1705
101	Sihao 4330	Sihong, Jiangsu, China	33.46	118.23	H1704
102	Sihao 4040	Sihong, Jiangsu, China	33.46	118.23	H1703
103	Yuedao 55	Vietnam	10.22	106.01	Y1A02409
104	Yanjing 9hao	Yancheng, Jiangsu, China	33.38	120.13	SS200707
105	Yangfujing 4901	Yangzhou, Jiangsu, China	32.24	119.26	SS200811
106	Zhengdao 18	Zhenzhou, Henan, China	34.76	113.65	GS2007033
107	Zhen9424	Zhenjiang, Jiangsu, China	32.12	119.27	ZD-05658
108	Nannongjing3786	Haerbin, Heilongjiang, China	44.04	125.42	
109	Yangfujing 8hao	Yancheng, Jiangsu, China	33.38	120.13	SS200608
110	Wuxiang99-8	Suihua, Heilongjiang, China	46.63	126.98	
111	Zhendao 99	Zhenjiang, Jiangsu, China	32.12	119.27	SS200106
112	Ningjing 2hao	Nanjing, Jiangsu, China	32.04	118.78	WPS05010476
113	Yuedao 107	Vietnam	10.22	106.01	Y1A02368
114	Yangfujing 7hao	Yangzhou, Jiangsu, China	32.24	119.26	SS200413
115	Wanqu 429bp	Haerbin, Heilongjiang, China	44.04	125.42	HS2013003
116	Xudao 3hao	Haerbin, Heilongjiang, China	34.26	117.2	SS200306
117	Xudao9201B	Xuzhou, Jiangsu, China	34.15	117.11	
118	Wuyunjing 21hao	Wujin, Jiangsu, China	31.78	119.95	SS200705-2
119	Zhongjing 131	Haerbin, Heilongjiang, China	44.04	125.42	H1620
120	Suyunuo	Haerbin, Heilongjiang, China	34.26	117.2	T832
121	Ebusinuodao	Suihua, Heilongjiang, China	46.63	126.98	T386
122	Yaxuenuo	Wuxian, Jiangsu, China	31.26	121.63	T480
123	Yuedao 108	Vietnam	10.22	106.01	Y1A02355
124	Luohanhuang	Jiangyin, Jiangsu, China	31.92	120.29	T560

125	Yuedao 41	Vietnam	10.22	106.01	Y1A02328	
126	Yuedao 43	Vietnam	10.22	106.01	Y1A02404	
127	Yuedao 48	Vietnam	10.22	106.01	Y1A02407	
128	Yuedao 49	Vietnam	10.22	106.01	Y1A02408	
129	Yuedao 61	Vietnam	10.22	106.01	Y1A02413	
130	Yuedao 9	Vietnam	10.22	106.01	Y1A02373	
131	Yuedao 13	Vietnam	10.22	106.01	Y1A02320	
132	Yuedao 22	Vietnam	10.22	106.01	Y1A02382	
133	Yuedao 50	Vietnam	10.22	106.01	Y1A02331	
134	Yuedao 62	Vietnam	10.22	106.01	Y1A02414	
135	Yuedao 66	Vietnam	10.22	106.01	Y1A02417	
136	Yuedao 109	Vietnam	10.22	106.01	Y1A02356	
137	IR112	Philippines	14.6	121	H1501	
138	IR64	Philippines	14.6	121	H1502	
139	Hainanxian R	Hainan, China	19.52	109.57	H1504	
140	Zajiaohaigu	Changjiang, Hainan, China	19.25	109.03	H1510	
141	Diantun502xuanzao	Kunming, Yunnan, China	25.04	102.73	ZD-05551	
142	Nongxiang 21	Changsha, Hunan, China	28.21	113	CNA200802496	
143	Nongxiang 25	Changsha, Hunan, China	28.21	113	GS2001021	
144	Fengyouwan 8hao	Changsha, Hunan, China	28.21	113	YS2009001	
145	Nongxiang26	Changsha, Hunan, China	28.21	113		
146	Xiangwanxian 17	Changsha, Hunan, China	28.21	113	XS2008035	
147	Yuzhenxiang	Changsha, Hunan, China	28.21	113	XS2009038	
148	Nuohangu	Kunming, Yunnan, China	25.04	102.73	H1434	
149	Lincangwazuhangu	Kunming, Yunnan, China	25.04	102.73	H1435	
150	Xiangxiandao 10hao	Changsha, Hunan, China	28.21	113	H1486	
151	LongtepuB	Fuzhou, Fujian, China	26.08	119.3	H1490	
152	Minghui63	Sanmingshi, Fujian, China	25.3	116.22		
153	Arias	Indonesia	6.08	94.45	H1339	
154	Yuetai B	Fogang, Guangdong, China	23.86	113.52	H1493	
155	Qimiaoxiang 2hao	Qingyuan, Guangdong, China	23.7	113.01	H1496	
156	Shengyou 2hao	Gaoyao, Guangdong, China	23.05	112.44	YS1994004	
157	II-32B	Changsha, Hunan, China	28.21	113	A0050	
158	CAMOR	Indonesia	6.08	94.45		10861
159	Gendjah Gempol	Indonesia	6.08	94.45		12483
160	Tijin	Japan	35.68	139.69	H1706	
161	Zhongguo 91	Japan	35.68	139.69	NL274	
162	Qiutianxiaoding	Japan	35.68	139.69	H1654	
163	M1004	Japan	35.68	139.69	Y1A01861	
164	Xiangchuanwuxinbaimi	Japan	35.68	139.69	H1655	
165	BULUH BAWU	Indonesia	6.08	94.45		16481
166	Hongnong 5hao	Wujiang, Jiangsu, China	31.16	120.63	T757	
167	Shenlenuo	Kunshan, Jiangsu, China	31.39	120.95	T691	
168	Yueguang	Japan	35.68	139.69	H1660	
169	Yimuhu	Japan	35.68	139.69	Y1A01857	
170	Qingkong	Nanjing, Jiangsu, China	32.04	118.78	Y1A01858	
171	RT61	Japan	35.68	139.69	Y1A01863	

172	IL38	Japan	35.68	139.69	ZD-05554
173	Jindao 1007	Dongli, Tianjin, China	39.14	117.13	GS2004043

Table S4. The parameters used for the PLINK software.

Step	Parameters	Note
Sequencing data filtering	plink --geno 0.2 --maf 0.05 --biallelic-only --vcf test.vcf --recode vcf-iid --out Test --allow-extra-chr	"—geno 0.2" sets the deletion rate of more than 20%, and "—maf 0.05" sets the deletion rate of less than 0.05 minor allele frequency
Retain unlinked SNP sites	plink --vcf Test.vcf --indep-pairwise 50 10 0.2 –out test.impute --allow-extra-chr	--Indep-pairwise 50 10 0.2"sets a 50kb window with a step size of 10 SNPs, and SNP correlation above 0.2
Extracting SNP sites	plink --vcf test.impute.vcf --extract test.impute.prune.in --recode vcf-iid --out test.impute.prune.in	"-- extract" extracts sites from filtered files
Format conversion	plink --vcf test.impute. prune.in.vcf --recode structure --out test.impute.prune.in	'-- recode structure' converts the filtered sites into structure format

Table S5. The parameters used for the Structure software.

Parameters
structure -m mainparams_structure_n.cfg -e extraparams -k n -i test.impute.prune.in.recode.strct_in -o Test_structure_k_n_1 -L 38498 -N 173 -D 1
structure -m mainparams_structure_n.cfg -e extraparams -k n -i test.impute.prune.in.recode.strct_in -o Test_structure_k_n _2 -L 38498 -N 173 -D 2
plink --vcf test.impute.vcf --extract test.impute.prune.in --recode vcf-iid --out test.impute.prune.in
structure -m mainparams_structure_n.cfg -e extraparams -k n -i test.impute.prune.in.recode.strct_in -o Test_structure_k_n _3 -L 38498 -N 173 -D 3

Set n=1-10 inferential subgroup numbers, "- L" to 38498 SNP sites, "- N" to 173 samples, and "- D" to 3 replicates.

Supplementary Figures

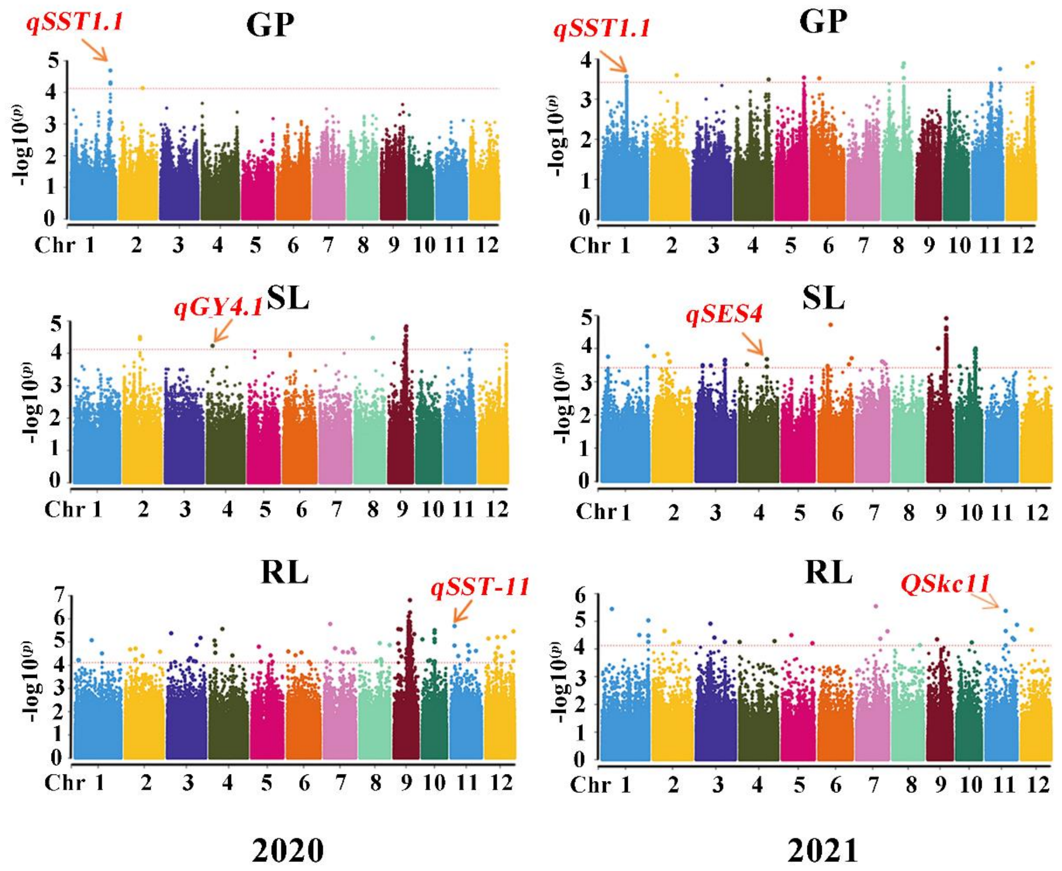

Figure S1. Manhattan plot of 173 rice germplasms obtained from GP, SL and RL salt tolerance germination experiments based on SNP markers (2020). Reported QTLs are indicated in the figure.

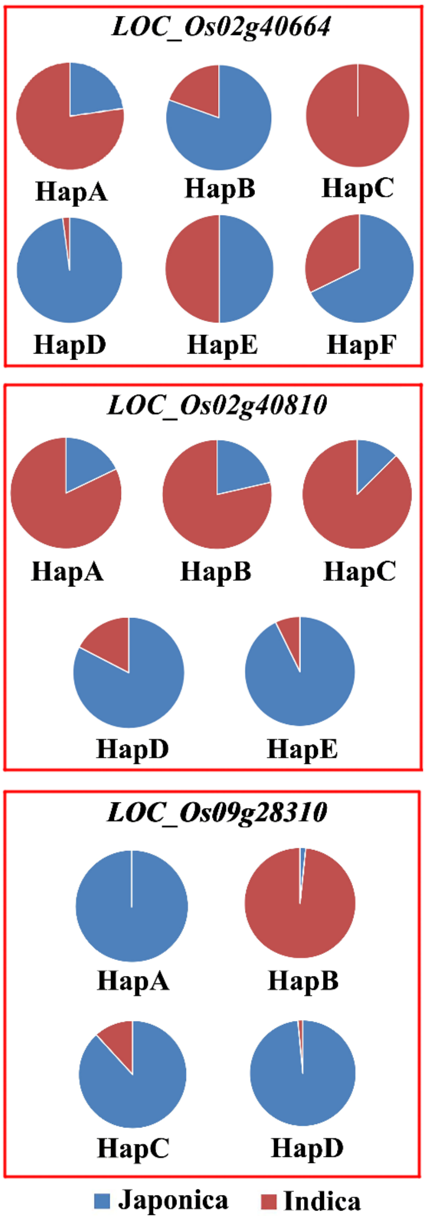

Figure S2. The distribution of candidate gene haplotypes in two subpopulations. The red area represents indica rice, and the blue area represents japonica rice.

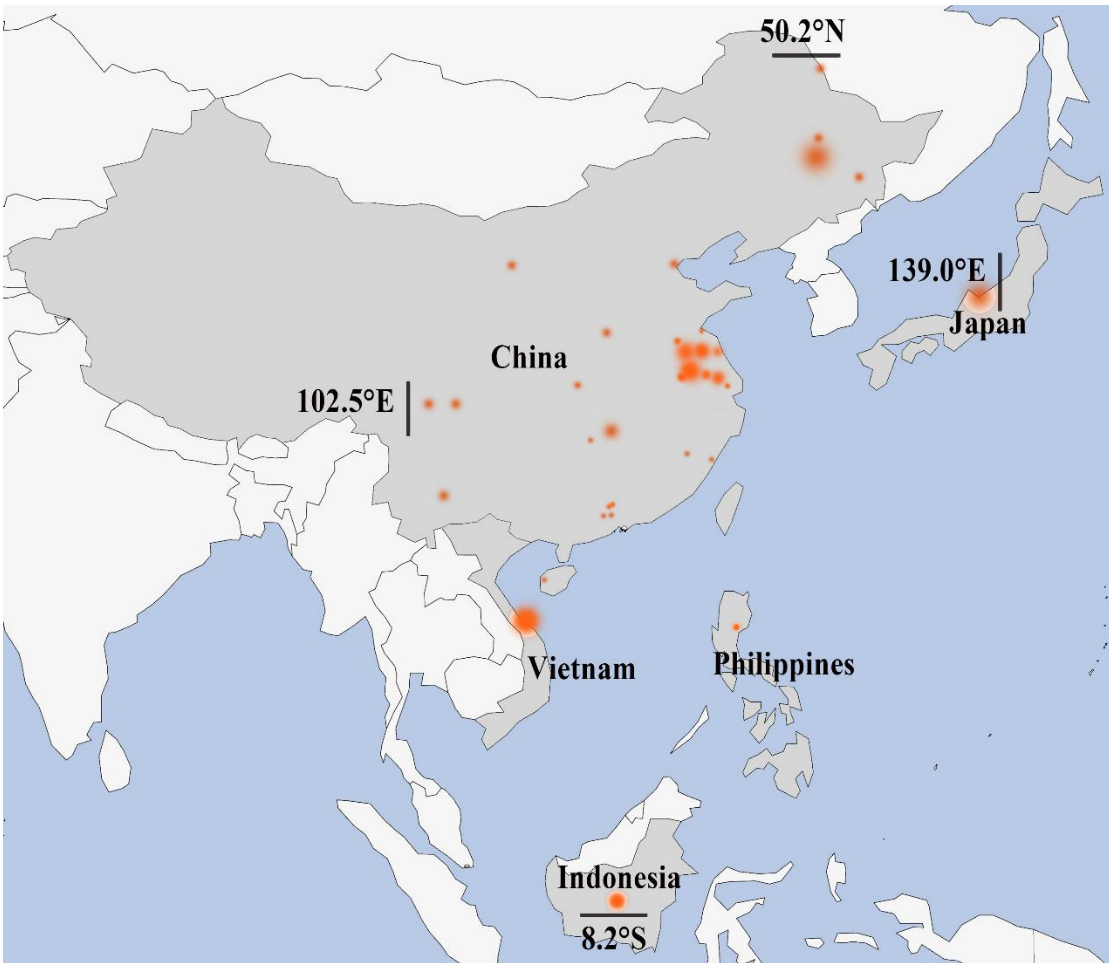

Figure S3. Geographical distribution of 173 rice varieties.
